# Supplementary material for: Chromatin Accessibility and Transcriptional Differences in Human Stem Cell-Derived Early-Stage Retinal Organoids
Source: Cells. 2022 Oct 28;11(21):3412. doi: 10.3390/cells11213412 (PMC9657268; doi:10.3390/cells11213412)
Supplement: Supplementary file 1 [file cells-11-03412-s001.zip › cells-1943944-supplementary.pdf]

Supplemental Figures. Chromatin accessibility and transcriptional differences in human stem cell-derived early-stage retinal organoids.

Melissa K. Jones[1], Devansh Agarwal[1,2], Kevin W. Mazo[1], Manan Chopra[1], Shawna L. Jurlina[1], Nicholas Dash[1], Qianlan Xu[1], Anna R. Ogata[1], Melissa Chow[1], Alex Hill[1], Netra K. Kambli[1,3], Guorong Xu[4], Roman Sasik[4], Amanda Birmingham[4], Kathleen M. Fisch[4,5], Robert N. Weinreb[1], Ray A. Enke[6], Dorota Skowronska-Krawczyk[1,7] and Karl J. Wahlin[1,*]

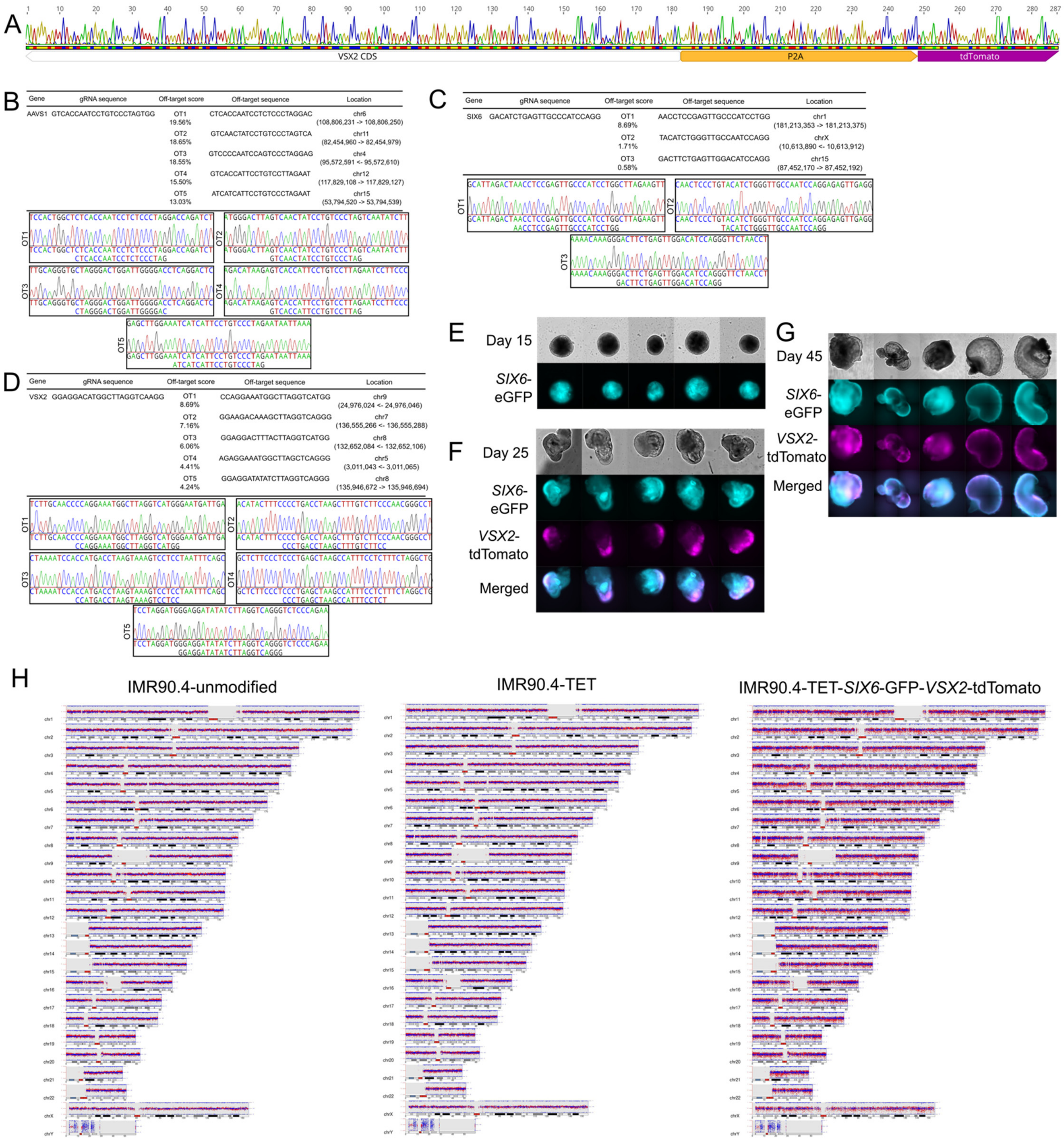

Figure S1. ***Validation of the SIX6-eGFP-VSX2-tdTomato fluorescent reporter into PSCs.*** (A) Sequence verification of homozygous insertion of the p2A-tdTomato cassette into the *VSX2* gene. PCR amplification of the *VSX2*-p2A-tdTomato cassette was followed by validation of homozygosity by DNA sequencing. (B-D) Off-target analysis following CRISPR-Cas9 gene editing. Off-target analysis of the top highest scoring regions of the genome for the AAVS1 (*PPRC1*), *SIX6*, and *VSX2* gRNAs detected no off-target mutations (B-D, respectively). (E) Copy number variation (CNV) analyses of unmodified IMR90.4 hiPSCs (E, top left) compared to IMR90.4 hiPSCs following CRISPR-Cas9 gene editing of the TET-

inducible platform (E, top right) and the *SIX6*-eGFP-*VSX2*-tdTomato reporters (E, bottom) with no obvious changes following gene editing. (F-H) Representative images of organoids during differentiation. (F) At day 15, *SIX6*-GFP is expressed throughout the organoid. (G) By day 25, persistent expression of GFP while regional *VSX2*-tdTomato expression was detected. Variability in organoid size and shape is due to manual dissection of optic vesicles. (H) By day 45, *SIX6*-GFP continues to be expressed throughout the organoids and *VSX2*-tdTomato expression expands to the edges of the entire organoid. Scale bars = 100 μm.

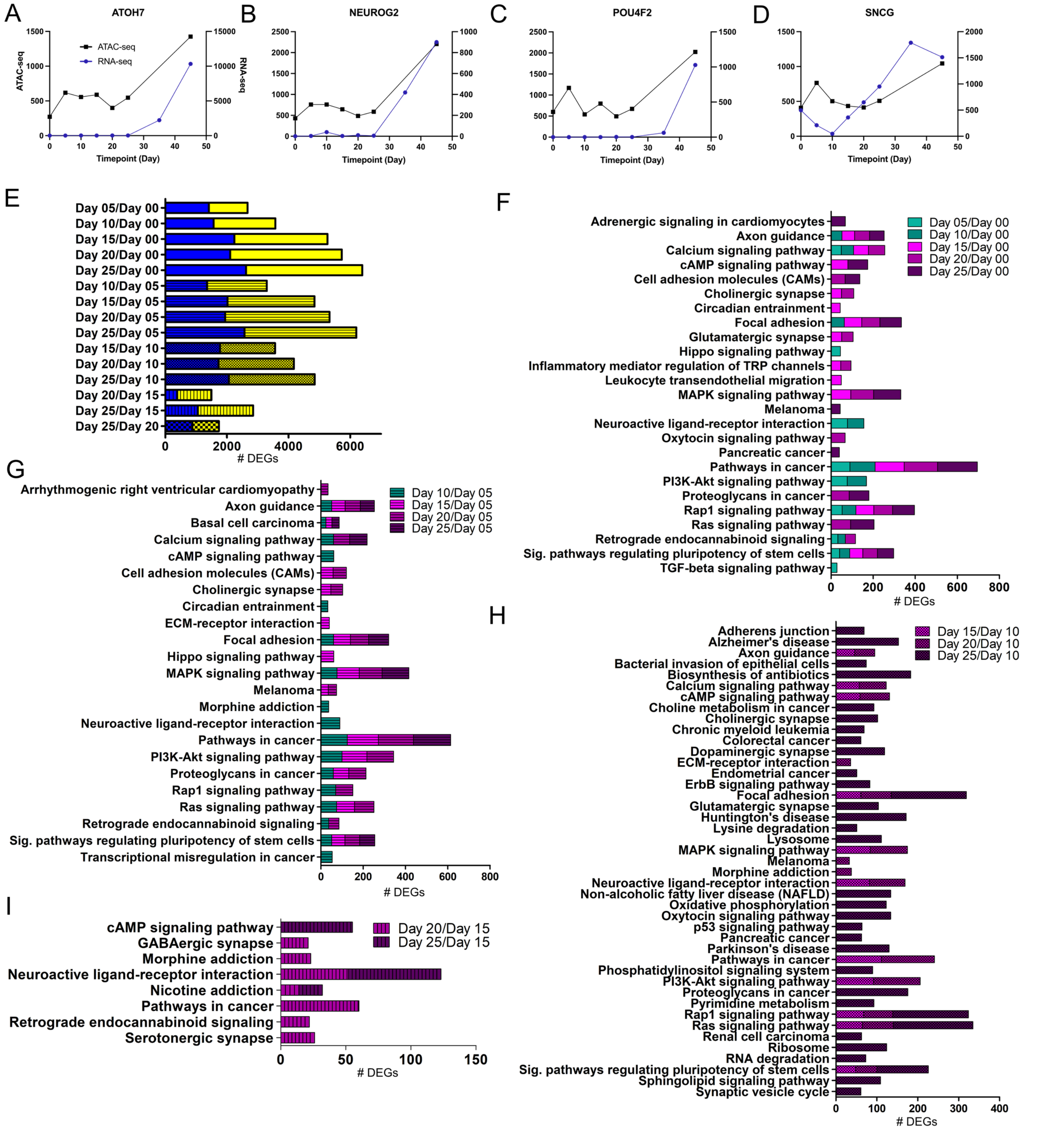

Figure S2. ***Analysis of DARs compared to expressed RNAs.*** (A-D) Selected DARs from ATAC-seq analysis were compared to their respective gene expression values from RNA-seq data. ATAC-seq peak heights are plotted on the left axis, RNA-seq gene expression values are plotted on the right axis. (E) Total number of DEGs for every time point comparison. (F-H) Significant pathways (FDR<0.001)) for every time point comparison as compared to day 00 (F), 05 (G), 10 (H), and 15 (I).

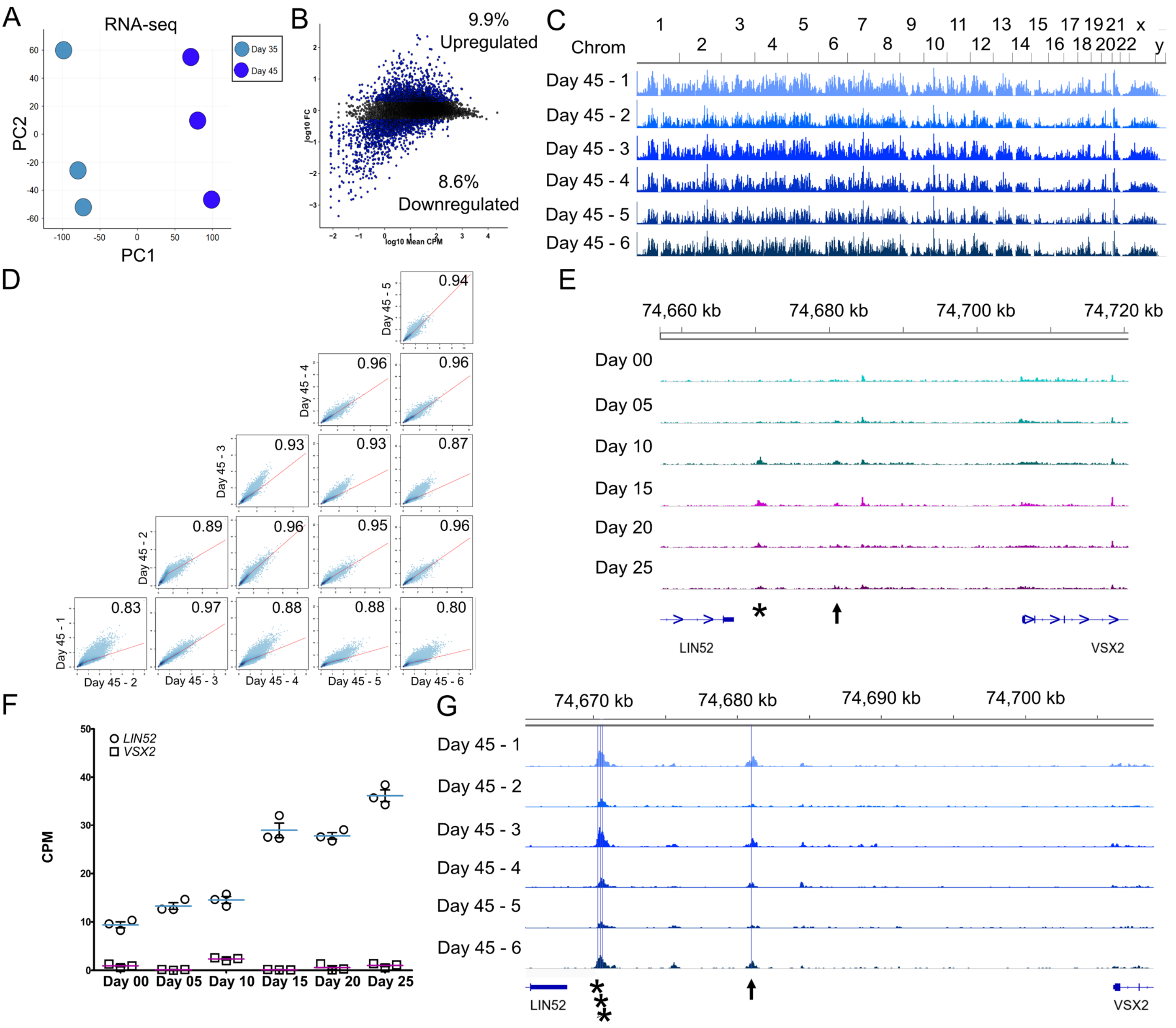

Figure S3. ***ATAC- and RNA-seq analysis of retinal organoids during retinal cell specification.*** Retinal organoids from days 35 and 45 were pooled (n=3 replicates; 8 organoids/replicate) for RNA-seq. (A) PCA of RNA-seq samples show variability between time points and replicates. (B) MA plot of expression of the 15,106 genes identified were compared and showed 9.9% of the genes (1,496 genes) were upregulated and 8.6% of the genes (1,294 genes) were downregulated at day 45 as compared to day 35. Individual day 45 organoids (n=6 replicates) were prepared for ATAC-seq analyses. (C) Genomic tracks for individual day 45 organoids indicate high similarity between replicates. (D) Pearson correlation of the individual organoid replicates (correlation coefficient = 0.80-0.97). (E) Genomic tracks of the intergenic region between *LIN52* and *VSX2* at days 00 to 25. The location of the published site (Diri et al., arrow) and the novel peak (asterisk) are less pronounced than at day 45. (F) Gene expression from RNA-seq analysis of *LIN52* and *VSX2* at day 45. CPM = counts per million. Scale for all genomic tracks = 0-100. (G) Genomic tracks of the intergenic region between *LIN52* and *VSX2* at day 45. A *VSX2* consensus binding sequence (TAATTAG) was detected at the same location as a previously published site (Diri et al., arrow) and three binding sequences were found in a novel peak downstream of *LIN52* (asterisks).

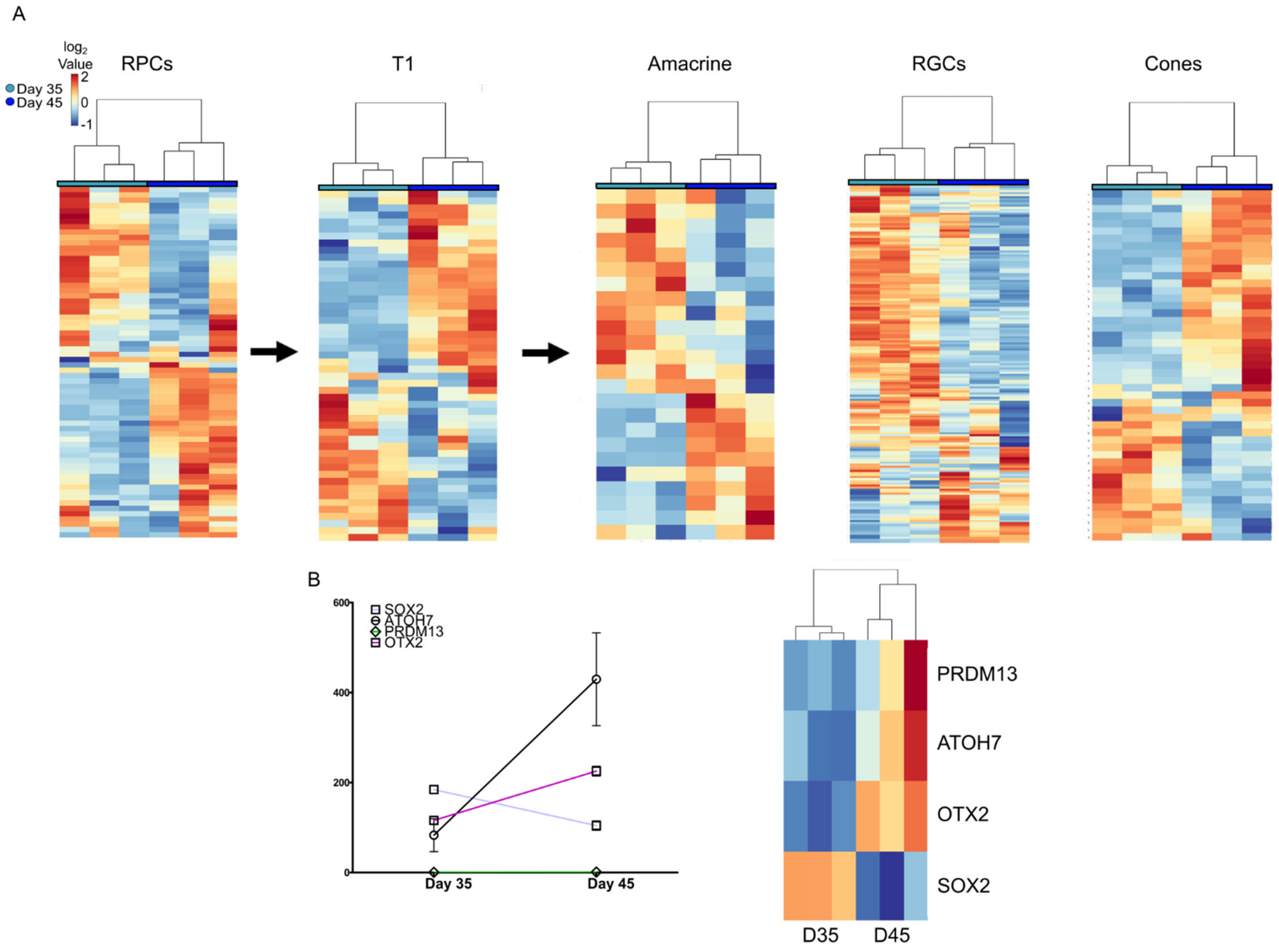

Figure S4: ***Comparison of gene expression in retinal organoids to fetal retinal tissue.*** Bulk RNA-seq from retinal organoids at days 35 and 45 were compared to a publicly available dataset of single cell RNA-seq (scRNA-seq) fetal retinal tissue at a comparable time-point in development (GEO: GSE142526; Sridhar et al., 2020). (A) Markers identified at different stages of development in the scRNA-seq of fetal retinal tissue (fetal day 59) were then used for expression comparison at days 35 and 45 bulk retinal organoid RNA-seq. RPCs = retinal progenitor cells, T1 = transition 1 stage cells, RGCs = retinal ganglion cells. (B) Characteristic gene markers for specific retinal cell types indicate increased expression of *PRDM13* (amacrine/transition 2 (T2) cells), *ATOH7* (T1 cells), and *OTX2* (photoreceptor/transition 3 (T3) cells) and decreased expression of *SOX2* (progenitor cells) at day 45.

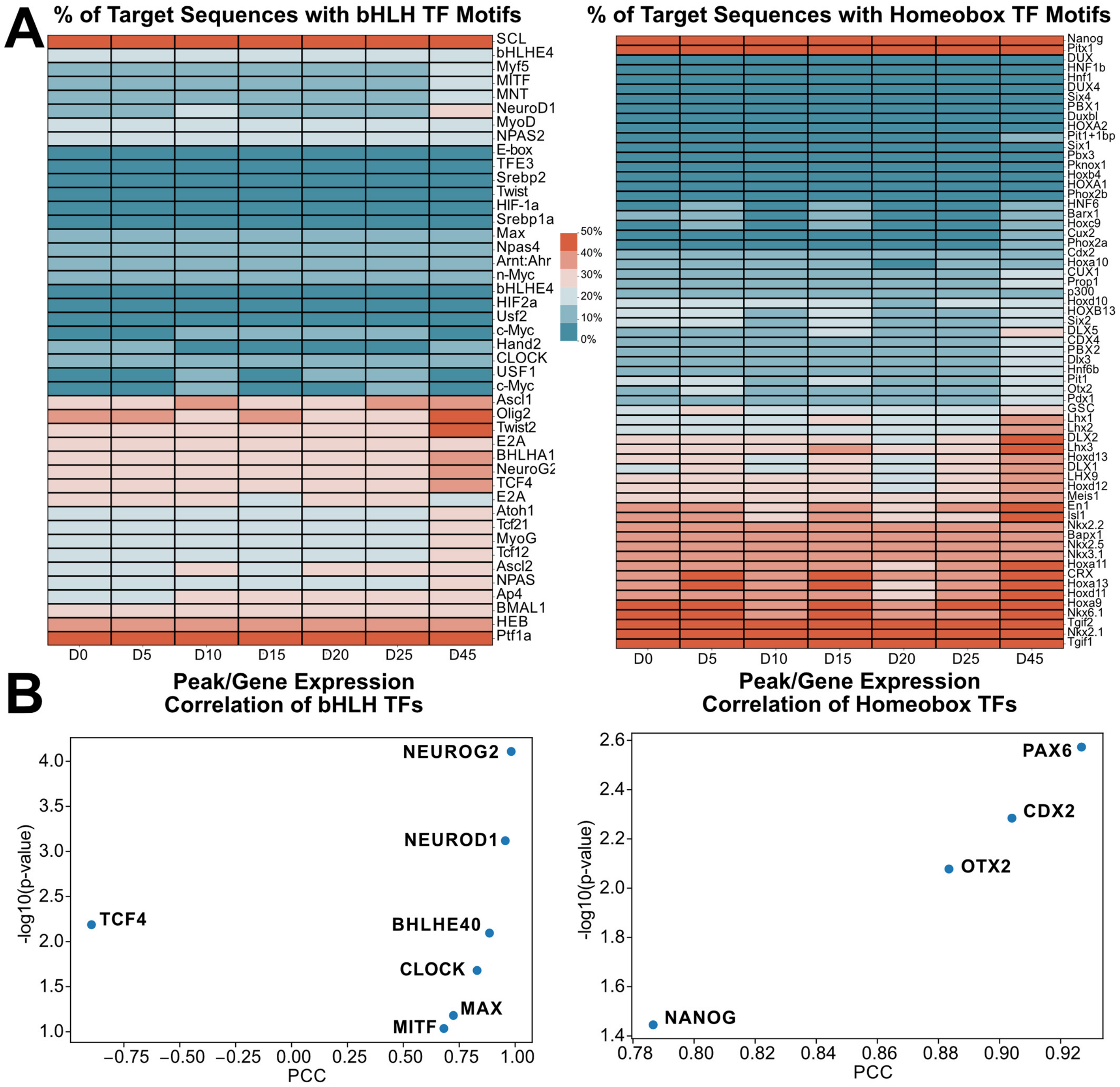

C

Table of Top Enriched Motifs in Each Family at Day 0

Zf	bHLH	Homeobox	HTH	bZIP	HMG	CCAAT
CTCF	SCL	OCT4-SOX2-TCF-NANOG	RFX	Bach2	Sox3	NFY
BORIS	Tcf12	Brn1	Rfx2	MafA	Sox10	
Sp5		Oct6	X-box	Jun-AP1	Sox21	
Zic3		Oct4	Rfx1	Fosl2	Sox2	
Sp2		Oct11	MYB	Fra2	Sox6	
Maz		En1	BMYB	Fos	Sox4	
Sp1		Six1			Sox17	
Zic2					Sox9	
Zfp281						
Zic						
KLF1						
ZNF467						
KLF14						
Klf9						
WT1						
KLF3						

Table of Top Enriched Motifs in Each Family at Day 45

Zf	bHLH	Homeobox	HTH	bZIP	HMG	CCAAT
CTCF	Atoh1	En1	Rfx2	JunB	Sox3	NF-E2
BORIS	NeuroD1	Lhx2	RFX	Fosl2	Sox10	
Zic3	BHLHA15	LHX9	X-box	Jun-AP1	Sox2	
Zic	NeuroG2	DLX2	Rfx1	Fos	Sox21	
Zic2	Twist2	Lhx3	BMYB	Fra1	Sox4	
Zfp281	TCF4	Lhx1	MYB	Atf3	Sox6	
Maz	SCL	DLX1	AMYB	Fra2	Sox9	
	Olig2	DLX5		BATF	Sox17	
	Ap4	Nkx6.1		AP-1		
	Tcf21	Dlx3		MafA		
	Ascl1	Isl1		Bach2		
	Tcf12	HNF6		Bach1		
	MyoG	Cux2		NF-E2		
	MyoD	Tgif2				
	Myf5	Nanog				
	Ptf1a	Tgif1				
		Hnf6b				
		Meis1				
		Otx2				
		CUX1				
		Pdx1				
		PAX6				
		Hoxa9				

Figure S5. ***Analysis of Enriched Motifs in each gene/TF Group.*** (A) Heatmaps of the ATAC-seq fold change for enriched bHLH and Homeobox TF motifs. Fold change was calculated from HOMER output using the number of target sequences

containing the motif divided by the number of background sequences containing the motif. (B) Scatterplots depicting -log10(p-value) versus Pearson correlation coefficient for motifs in each TF/gene group. Coefficients were generated by correlating the temporal (day 0-day 45) trend of gene expression from RNA-seq to the trend of open chromatin peaks from ATAC-seq. (C) Tables listing the genes plotted in the respective day 00 and day 45 swarm plots in **Fig 5D**, grouped by gene type.

Table S1. ***Primer sequences for amplification and sequencing of the AAVS1 (PPRC1), SIX6, and VSX2 gRNA top off-target sites***. Primers for amplification (F, rev) of each off-target (OT) site for the top 5 AAVS1 (*PPRC1*) gRNA targeting sites (**Fig. S1B**), the only 3 identified *SIX6* gRNA targeting sites (**Fig. S1C**), and the top 5 *VSX2* gRNA targeting sites (**Fig. S1D**) and for DNA sequencing (Seq) of the amplicon.

Target	Description	Primer sequence
AAVS1 off-target site 1	AAVS1_893_OT1_F	ACTTTGGGAGGCTGAGGCAAGAAGATTG
	AAVS1_893_OT1_Rev	TCTTCCTTCTGAACTCTTCCCACAGGGATT
	AAVS1_Seq_OT1	TTCCCACTTCCTCATTTCTACTTC
AAVS1 off-target site 2	AAVS1_904_OT2_F	ACCCTCACCAGCACTGGAAATTGAAAA
	AAVS1_904_OT2_Rev	TTGGGTTTCTCTCCTCTAGCATGGATCTC
	AAVS1_Seq_OT2	CCCATACTTTCTTGGGTAATGTTC
AAVS1 off-target site 3	AAVS1_861_OT3_F	CCAAGGGCTCTTCAGTCAGGCTCTTACA
	AAVS1_861_OT3_Rev	ATGGAGGGAGCATTTGGACCAGACCTA
	AAVS1_Seq_OT3	AATGCTGTTCAAGAGCTAAGTCCT
AAVS1 off-target site 4	AAVS1_890_OT4_F	TGTCCTGAGTTACAAGGGTGGACAGCAC
	AAVS1_890_OT4_Rev	TTCTTTGTGCCAAGCCCCATGTTAGGTA
	AAVS1_Seq_OT4	TGGAGAAGTCTAGTGCTCACATTC
AAVS1 off-target site 5	AAVS1_876_OT5_F	TGGTGCAATCATGGCTCACTACAGCTTC
	AAVS1_876_OT5_Rev	TCCCTTCCCCCAGGTTAGATAATGTTTTGG
	AAVS1_Seq_OT5	AGTAGAGGCGAGGTCTCACTATGT
SIX6 off-target site 1	SIX6_713_OT1_F	GCAGTGGGCAAACTGCCTCCA
	SIX6_713_OT1_Rev	GCTCCCTCCTGCTCTTCCTCACC
	SIX6_Seq_OT1	AGGGCAGGGAATAAGCAAAT
SIX6 off-target site 2	SIX6_803_OT2_F	GAGACTCCAGTGCAGCCACATGGT
	SIX6_803_OT2_Rev	TGGCAGTGTGGTTCGGGGAGT
	SIX6_Seq_OT2	GCTGAATTGGTCACAGCTCA
SIX6 off-target site 3	SIX6_812_OT3_F	TGTCTTTTGGCTTTCAGGCATGTGG
	SIX6_812_OT3_Rev	AGGGCAGCTTCCAACAGAGAGCAAG
	SIX6_Seq_OT3	CATAAGCCAAAAGGCTGCTT
VSX2 off-target site 1	VSX2_1181_OT1_F	GAAGGAAAGGCAGAGGCTGTTTCCAGA
	VSX2_1181_OT1_Rev	GGCATAATGCAACTGACACGCATTCAC
	VSX2_Seq_OT1	TAATGCCTATTATGAGGGACAAGG
VSX2 off-target site 2	VSX2_873_OT2_F	GGCTGTTCTCTGACAGAATTGGAAGCA
	VSX2_873_OT2_Rev	TGTCAAGGGCCTGAGATCCCCTCATAC
	VSX2_Seq_OT2	AGCTAGCCTGAATCTTCTTCCATA
VSX2 off-target site 3	VSX2_951_OT3_F	CCTGCAGTTTGCCAAGCTCAAAAAGAC
	VSX2_951_OT3_Rev	TCTGATTGCGTCATTCCTGTGGTGTGT
	VSX2_Seq_OT3	GCTGCCCTCTAGTGGTACATAAAT
VSX2 off-target site 4	VSX2_821_OT4_F	AGTCAGGACCCAATTTTCTCGGTGAGC
	VSX2_821_OT4_Rev	AGCTGCCTTTACAGACCGAAGGACAGG
	VSX2_Seq_OT4	CAGGAGTTAAAGGGAATGACAAGT
VSX2 off-target site 5	VSX2_964_OT5_F	GTGGGGAAAAAGCAGCAGTGTTCACCT
	VSX2_964_OT5_Rev	TATTGGTATCCCCACCCAGACCACCTC
	VSX2_Seq_OT5	CTGAGACCAGGAAGTACAACAAGA

Table S2: ***Top 100 most highly differentially expressed genes (DEGs) from day 25/day 00.*** Log_2 fold change (FC) was calculated from the CPM averages at day 25 and day 00, and CPM averages at each time point were used for hierarchical cluster analysis (**Fig. 2E**).

ENSEMBL	ENTREZID	SYMBOL	logFC	AveExpr	t	P.Value	adj.P.Val
ENSG00000092068	23428	SLC7A8	-5.4552672	6.23379162	-61.742569	1.40E-25	2.02E-21
ENSG00000088305	1789	DNMT3B	-5.5525949	7.79940069	-55.748504	1.18E-24	8.55E-21
ENSG00000128567	5420	PODXL	-4.1111268	8.07030437	-48.121995	2.56E-23	8.04E-20
ENSG00000086475	22929	SEPHS1	-3.1751428	8.21893831	-48.961364	1.78E-23	8.04E-20
ENSG00000185559	8788	DLK1	11.2480714	10.3228784	47.9828057	2.78E-23	8.04E-20
ENSG00000166165	1152	CKB	3.41865822	10.0205022	45.9721651	6.63E-23	1.60E-19
ENSG00000184697	9074	CLDN6	-6.3048487	6.71956051	-43.05703	2.60E-22	4.42E-19
ENSG00000170421	3856	KRT8	-4.1487814	7.3996672	-42.936353	2.75E-22	4.42E-19
ENSG00000182670	7267	TTC3	3.5110893	7.62942726	43.4427702	2.16E-22	4.42E-19
ENSG00000026025	7431	VIM	5.55587698	9.4746477	42.557175	3.31E-22	4.79E-19
ENSG00000111057	3875	KRT18	-5.612253	5.79946183	-37.780488	3.94E-21	5.17E-18
ENSG00000109472	1363	CPE	3.96031919	7.00081066	37.4624053	4.69E-21	5.65E-18
ENSG00000112759	2030	SLC29A1	-3.4892569	6.74379692	-37.258663	5.25E-21	5.84E-18
ENSG00000103044	3038	HAS3	-4.9867465	4.79193969	-36.925192	6.33E-21	6.10E-18
ENSG00000102096	11040	PIM2	-3.5021214	5.73682075	-36.997036	6.08E-21	6.10E-18
ENSG00000147257	2719	GPC3	3.48479663	9.98160142	36.5914178	7.64E-21	6.50E-18
ENSG00000065320	9423	NTN1	4.71173468	6.08871647	36.6573463	7.36E-21	6.50E-18
ENSG00000121570	55211	DPPA4	-4.0453407	6.95787894	-36.397862	8.53E-21	6.85E-18
ENSG00000156453	5097	PCDH1	-4.2304364	6.77352597	-36.108221	1.01E-20	7.66E-18
ENSG00000106484	4232	MEST	3.23143978	9.49256247	35.9057434	1.13E-20	8.18E-18
ENSG00000122756	1271	CNTFR	4.70074875	6.17630663	35.3916505	1.53E-20	1.05E-17
ENSG00000090097	57060	PCBP4	3.62424204	7.15398726	35.2547884	1.65E-20	1.09E-17
ENSG00000109861	1075	CTSC	-3.5684122	6.350168	-34.841073	2.11E-20	1.33E-17
ENSG00000010278	928	CD9	-4.7052747	4.02368089	-34.573448	2.48E-20	1.47E-17
ENSG00000132718	23208	SYT11	3.67769351	7.43529654	34.5316995	2.54E-20	1.47E-17
ENSG00000158769	50848	F11R	-3.4609105	6.15923288	-34.419899	2.71E-20	1.51E-17
ENSG00000114115	5947	RBP1	4.56794676	5.97950784	34.2902234	2.94E-20	1.57E-17
ENSG00000100604	1113	CHGA	-4.5030081	4.51744183	-33.629818	4.39E-20	2.27E-17
ENSG00000182095	84629	TNRC18	4.35188782	6.79953312	33.5381469	4.64E-20	2.32E-17
ENSG00000108604	6603	SMARCD2	-3.7712193	6.01361799	-33.340734	5.25E-20	2.53E-17
ENSG00000166848	54386	TERF2IP	3.64367202	5.80935294	33.082778	6.16E-20	2.87E-17
ENSG00000157851	56896	DPYSL5	3.55969828	8.28279593	32.8464466	7.15E-20	3.23E-17
ENSG00000115461	3488	IGFBP5	5.46355469	7.97697383	32.5457296	8.65E-20	3.79E-17
ENSG00000104332	6422	SFRP1	-2.768797	9.82074067	-32.464672	9.10E-20	3.87E-17
ENSG00000162878	91461	PKDCC	-4.8812458	6.77423823	-32.418709	9.37E-20	3.87E-17
ENSG00000166831	348093	RBPMS2	-3.0054786	5.95190433	-32.15602	1.11E-19	4.45E-17
ENSG00000158246	115572	FAM46B	-4.7307367	4.71273515	-32.051357	1.19E-19	4.51E-17
ENSG00000154096	7070	THY1	-3.4014392	6.4537077	-32.086714	1.16E-19	4.51E-17
ENSG00000170558	1000	CDH2	3.31535869	7.8440603	32.0096399	1.22E-19	4.51E-17

ENSG00000143494	79805	VASH2	-4.441216	4.28714786	-31.791621	1.40E-19	5.07E-17
ENSG00000104635	23516	SLC39A14	-3.015478	6.31934017	-31.735741	1.45E-19	5.13E-17
ENSG00000148200	2649	NR6A1	-5.5983555	7.23950169	-31.572024	1.62E-19	5.57E-17
ENSG00000240563	54596	L1TD1	-9.1265859	4.4950123	-31.535278	1.67E-19	5.63E-17
ENSG00000206557	131405	TRIM71	-4.9947761	7.26598927	-31.37203	1.84E-19	5.86E-17
ENSG00000110042	23220	DTX4	3.61845352	7.66054807	31.3557371	1.86E-19	5.86E-17
ENSG00000240694	10687	PNMA2	4.54886234	5.29112824	31.4025563	1.81E-19	5.86E-17
ENSG00000105699	51599	LSR	-4.5596068	6.3611626	-31.308436	1.92E-19	5.92E-17
ENSG00000101144	655	BMP7	5.70255141	6.45602886	30.9942985	2.37E-19	7.14E-17
ENSG00000018189	22902	RUFY3	3.44192052	6.08014875	30.9252693	2.48E-19	7.31E-17
ENSG00000182580	2049	EPHB3	3.10759685	6.21684447	30.7701516	2.75E-19	7.94E-17
ENSG00000167614	57348	TTYH1	3.68768189	8.2350584	30.741954	2.80E-19	7.94E-17
ENSG00000164638	222962	SLC29A4	3.02659927	6.14094382	30.2729943	3.85E-19	1.07E-16
ENSG00000102144	5230	PGK1	-2.337599	9.19332871	-30.099658	4.33E-19	1.18E-16
ENSG00000136160	1910	EDNRB	3.21050399	6.05411681	29.9258063	4.88E-19	1.31E-16
ENSG00000171314	5223	PGAM1	-2.4875593	9.7333132	-29.880633	5.03E-19	1.32E-16
ENSG00000156966	93010	B3GNT7	-5.6154868	3.37834414	-29.750106	5.51E-19	1.42E-16
ENSG00000182175	56963	RGMA	2.74528681	7.98303634	29.6633752	5.85E-19	1.48E-16
ENSG00000205336	9289	ADGRG1	5.39057684	6.37136169	29.5767426	6.21E-19	1.55E-16
ENSG00000147601	7013	TERF1	-3.0008604	6.85828931	-29.514304	6.49E-19	1.59E-16
ENSG00000166073	11245	GPR176	-4.8657655	5.40451548	-29.33512	7.35E-19	1.77E-16
ENSG00000141736	2064	ERBB2	-2.432454	8.58032029	-29.189091	8.15E-19	1.93E-16
ENSG00000148204	286204	CRB2	3.34455907	7.36541411	29.0489574	9.00E-19	2.06E-16
ENSG00000149294	4684	NCAM1	4.92851622	6.55488329	29.056657	8.95E-19	2.06E-16
ENSG00000163251	7855	FZD5	4.58410982	8.9116679	28.9755305	9.48E-19	2.14E-16
ENSG00000167600	29785	CYP2S1	-5.7810411	4.27331092	-28.872671	1.02E-18	2.27E-16
ENSG00000050344	9603	NFE2L3	-4.4991009	4.45714902	-28.81306	1.06E-18	2.30E-16
ENSG00000165349	84889	SLC7A3	-3.0521953	5.7199954	-28.832231	1.05E-18	2.30E-16
ENSG00000185885	8519	IFITM1	-4.919477	4.29507432	-28.671457	1.18E-18	2.50E-16
ENSG00000011028	9902	MRC2	3.28735537	6.73245948	28.5606927	1.27E-18	2.67E-16
ENSG00000035403	7414	VCL	-3.4313913	7.84926296	-28.450508	1.38E-18	2.78E-16
ENSG00000137166	116113	FOXP4	3.13924108	6.57988214	28.4594011	1.37E-18	2.78E-16
ENSG00000114315	3280	HES1	3.37741452	6.27679713	28.4440876	1.39E-18	2.78E-16
ENSG00000171533	4135	MAP6	5.34777692	5.87878957	28.2416638	1.61E-18	3.18E-16
ENSG00000204531	5460	POU5F1	-10.796042	4.11895534	-27.925012	2.07E-18	4.03E-16
ENSG00000186469	54331	GNG2	3.57661789	6.41857632	27.880731	2.09E-18	4.03E-16
ENSG00000140545	4240	MFGE8	-2.546286	7.98834562	-27.764335	2.28E-18	4.33E-16
ENSG00000078018	4133	MAP2	4.40588666	6.8418825	27.7311479	2.33E-18	4.38E-16
ENSG00000131389	6533	SLC6A6	-3.7122395	5.94690911	-27.626583	2.52E-18	4.61E-16
ENSG00000188042	10123	ARL4C	2.97954146	7.43007175	27.6369463	2.50E-18	4.61E-16
ENSG00000139219	1280	COL2A1	3.58246036	7.06296235	27.4244657	2.93E-18	5.30E-16
ENSG00000091129	4897	NRCAM	6.05658109	6.30204641	27.4065142	2.97E-18	5.31E-16
ENSG00000184613	4753	NELL2	2.89570034	7.70134459	27.3758188	3.04E-18	5.36E-16

ENSG00000183044	18	ABAT	5.75595152	5.79677779	26.8239871	4.62E-18	8.04E-16
ENSG00000141622	494470	RNF165	3.27029036	5.65723091	26.7294733	4.96E-18	8.54E-16
ENSG00000155966	2334	AFF2	3.97715728	6.17594523	26.6541585	5.26E-18	8.94E-16
ENSG00000085117	3732	CD82	3.27377525	4.61999391	26.6163655	5.41E-18	9.09E-16
ENSG00000065717	7089	TLE2	4.08536807	4.4062528	26.5673975	5.62E-18	9.34E-16
ENSG00000168772	80319	CXXC4	4.46964926	4.72538107	26.2228334	7.34E-18	1.21E-15
ENSG00000253305	56100	PCDHGB6	3.16463571	7.16885227	26.1656136	7.67E-18	1.25E-15
ENSG00000135097	4440	MSI1	2.46910195	7.5906436	25.9030649	9.43E-18	1.50E-15
ENSG00000126016	154796	AMOT	3.46477048	7.44725444	25.9108083	9.37E-18	1.50E-15
ENSG00000111913	9750	RIPOR2	-4.5198352	1.7711294	-25.763003	1.05E-17	1.66E-15
ENSG00000162551	249	ALPL	-2.8195523	7.22880915	-25.738217	1.07E-17	1.67E-15
ENSG00000077279	1641	DCX	5.4051844	6.01279144	25.7234627	1.09E-17	1.67E-15
ENSG00000127418	53834	FGFRL1	3.26417061	5.95808224	25.6149468	1.19E-17	1.80E-15
ENSG00000105270	25999	CLIP3	3.22045227	7.11556904	25.6015791	1.20E-17	1.80E-15
ENSG00000129946	25759	SHC2	4.923798	5.17927527	25.4264718	1.38E-17	2.05E-15
ENSG00000150510	220108	FAM124A	-3.4353321	4.76935015	-25.352045	1.46E-17	2.16E-15
ENSG00000160360	26086	GPSM1	2.68487328	6.5102879	25.1421719	1.73E-17	2.53E-15
ENSG00000172379	9915	ARNT2	3.60723782	6.11909782	25.0289634	1.90E-17	2.75E-15

Table S3: ***Pathway analysis of genes identified from DARs and DEGs at day 15/00 and day 25/00 comparison.*** The 110 genes from the DAR+DEG comparison of day15/day00 (**Table S4**) were input into DAVID Bioinformatics Resources (v6.8). Pathway analysis yielded 677 pathways/keywords/terms; 9 pathways met significance (False discovery rate (FDR)<0.1). Amongst 669 genes from the day 25/day 00 dataset, 1487 pathways were identified, 68 met significance. Count refers to number of genes from dataset found in pathway, % genes from list refers to percent of genes in pathway from dataset provided.

**Day 15/Day 00**				
**Term**	**Count**	**%**	**P-Value**	**FDR**
GO:0030054~cell junction	15	3.65853659	1.68E-04	0.02109417
GO:0043235~receptor complex	14	3.41463415	3.06E-04	0.02690028
GO:0016324~apical plasma membrane	19	4.63414634	3.57E-04	0.02690028
GO:0005737~cytoplasm	138	33.6585366	4.67E-04	0.02936461
GO:0043005~neuron projection	18	4.3902439	1.11E-03	0.05248825
GO:0042803~protein homodimerization activity	30	7.31707317	3.22E-04	0.06777277
GO:0007165~signal transduction	46	11.2195122	6.26E-05	0.09709172
GO:0098609~cell-cell adhesion	14	3.41463415	8.90E-05	0.09709172
GO:0007399~nervous system development	21	5.12195122	1.81E-04	0.09881239

**Day 25/Day 00**				
**Term**	**Count**	**%**	**P-Value**	**FDR**
GO:0007399~nervous system development	52	5.40540541	6.93E-11	2.59E-07
KW-0524~Neurogenesis	39	4.05405405	1.31E-08	1.53E-06
REGION:Disordered	707	73.4927235	7.49E-10	2.95E-06
GO:0043025~neuronal cell body	45	4.67775468	6.96E-08	2.03E-05
GO:0005737~cytoplasm	315	32.7442827	1.20E-06	2.34E-04
hsa04015:Rap1 signaling pathway	29	3.01455301	1.73E-06	5.22E-04
KW-0677~Repeat	299	31.0810811	3.32E-05	8.96E-04
GO:0007411~axon guidance	26	2.7027027	7.44E-07	0.00118915
GO:0050770~regulation of axonogenesis	9	0.93555094	1.27E-06	0.00118915
GO:0045892~negative regulation of transcription, DNA-templated	53	5.50935551	3.12E-06	0.00194646
GO:0009887~animal organ morphogenesis	20	2.07900208	1.39E-05	0.00740914
GO:0010628~positive regulation of gene expression	46	4.78170478	1.62E-05	0.00744056
GO:0045930~negative regulation of mitotic cell cycle	10	1.03950104	1.79E-05	0.00744056
GO:0001657~ureteric bud development	10	1.03950104	2.29E-05	0.00839521
GO:0016358~dendrite development	11	1.14345114	2.47E-05	0.00839521
GO:0042383~sarcolemma	16	1.66320166	7.69E-05	0.00896627
GO:0043005~neuron projection	35	3.63825364	1.06E-04	0.00951328
GO:0030425~dendrite	40	4.15800416	1.14E-04	0.00951328
GO:0000122~negative regulation of transcription from RNA polymerase II promoter	72	7.48440748	3.41E-05	0.01007871
GO:0043410~positive regulation of MAPK cascade	21	2.18295218	3.50E-05	0.01007871
GO:0045121~membrane raft	26	2.7027027	1.71E-04	0.01197894
GO:0014069~postsynaptic density	27	2.80665281	1.85E-04	0.01197894
KW-0333~Golgi apparatus	67	6.96465696	0.00109243	0.01342041
GO:0030018~Z disc	17	1.76715177	3.40E-04	0.01534126
GO:0005634~nucleus	314	32.6403326	3.41E-04	0.01534126

GO:0005794~Golgi apparatus	76	7.9002079	3.63E-04	0.01534126
GO:0016328~lateral plasma membrane	12	1.24740125	3.91E-04	0.01534126
GO:0005911~cell-cell junction	21	2.18295218	4.16E-04	0.01534126
GO:0016327~apicolateral plasma membrane	7	0.72765073	4.21E-04	0.01534126
KW-0966~Cell projection	76	7.9002079	0.00245746	0.01597347
KW-0206~Cytoskeleton	85	8.83575884	0.00315925	0.01760154
GO:0008284~positive regulation of cell proliferation	46	4.78170478	8.25E-05	0.02083618
GO:0045893~positive regulation of transcription, DNA-templated	56	5.82120582	8.35E-05	0.02083618
GO:0007420~brain development	28	2.91060291	9.48E-05	0.02216769
GO:0008285~negative regulation of cell proliferation	40	4.15800416	1.16E-04	0.02560606
hsa04520:Adherens junction	13	1.35135135	1.82E-04	0.0275088
GO:0007626~locomotory behavior	14	1.45530146	1.33E-04	0.02774001
GO:0005912~adherens junction	19	1.97505198	9.18E-04	0.02973589
KW-0963~Cytoplasm	292	30.3534304	0.00654499	0.03190682
GO:0007409~axonogenesis	14	1.45530146	1.69E-04	0.03324197
GO:0051897~positive regulation of protein kinase B signaling	17	1.76715177	2.06E-04	0.03863194
GO:0001725~stress fiber	11	1.14345114	0.00151087	0.040895
GO:0099060~integral component of postsynaptic specialization membrane	7	0.72765073	0.00157415	0.040895
GO:0098685~Schaffer collateral - CA1 synapse	12	1.24740125	0.00174175	0.040895
GO:0009925~basal plasma membrane	9	0.93555094	0.00183229	0.040895
GO:0098982~GABA-ergic synapse	11	1.14345114	0.00185999	0.040895
GO:0030054~cell junction	22	2.28690229	0.00186405	0.040895
hsa04514:Cell adhesion molecules	19	1.97505198	8.20E-04	0.04124969
GO:0030335~positive regulation of cell migration	26	2.7027027	2.46E-04	0.0438168
GO:0060644~mammary gland epithelial cell differentiation	6	0.62370062	2.61E-04	0.04433558
hsa05200:Pathways in cancer	44	4.57380457	0.00106119	0.04578297
KW-0597~Phosphoprotein	468	48.6486486	0.00209154	0.0606548
GO:0098978~glutamatergic synapse	30	3.11850312	0.0030307	0.06224135
GO:0015629~actin cytoskeleton	23	2.39085239	0.00309605	0.06224135
GO:0048013~ephrin receptor signaling pathway	10	1.03950104	4.04E-04	0.06234644
GO:0051965~positive regulation of synapse assembly	11	1.14345114	4.16E-04	0.06234644
KW-0037~Angiogenesis	17	1.76715177	0.00106989	0.06258856
GO:0005769~early endosome	26	2.7027027	0.00325901	0.06333343
GO:0001578~microtubule bundle formation	8	0.83160083	4.59E-04	0.06604663
GO:0043197~dendritic spine	17	1.76715177	0.00375588	0.06842747
KW-0010~Activator	54	5.61330561	0.00378806	0.06970034
GO:0060076~excitatory synapse	7	0.72765073	0.00427949	0.0756044
GO:0048471~perinuclear region of cytoplasm	51	5.3014553	0.00490769	0.08415237
GO:0009986~cell surface	44	4.57380457	0.00534085	0.08745433
COMPBIAS:Polar residues	369	38.3575884	8.90E-05	0.08749784
hsa04510:Focal adhesion	21	2.18295218	0.00249744	0.09427839
hsa04151:PI3K-Akt signaling pathway	31	3.22245322	0.00313431	0.0995326
hsa04010:MAPK signaling pathway	27	2.80665281	0.00329578	0.0995326

Table S4: ***Genes identified from the comparison of DARs and expressed RNA (DEGs) for pathway analysis.*** Unique genes from the day 15/day 00, day 25/day 00, and common genes between the two comparisons (**Figure 2I**).

**Day 25/Day 00**	**Day 15/Day 00**	**Common**
CDH2	CDH3	RASA4B
ZNF737	PATJ	TOM1L1
SHISAL2B	CPQ	YAF2
ERVMER34-1	TMSB15A	CHST4
MAMLD1	HIBADH	GDF11
OCLN	KIF12	CNKSR1
FPGT-TNNI3K	ZBED9	PRSS16
ATP9A	ZNF618	CDS1
GPC6	C8orf34	ZBTB18
MBNL2	CLCN5	TRIM38
LHFPL2	ADD2	VAX1
CALCRL	KDF1	DEPP1
TRIB1	ARHGEF19	CHD3
CDKN1B	CPE	FAM107A
CDKN1C	CCM2L	CELF3
BCKDK	TRPM6	CHN1
APC2	C14orf37	CAVIN3
APBB3	TTL	HOGA1
B3GALT5	TTC30B	MAL2
RRAGB	IGSF11	ARL11
TRIM22	ZDHHC15	ZNF641
TUBB4A	DNAH10	TC2N
RAPGEF3	GRAMD2A	KLHDC7A
VAV3	ENPEP	GOLT1A
TOMM40	SLC29A1	TMEM125
BAIAP2	ETS1	PROKR2
CAP2	PLA2R1	ICA1L
SEMA4F	EXPH5	ZFP42
NEBL	SEPT8	CPEB2
SLC34A2	SLC44A1	UGT3A1
IGF2BP3	CD2AP	ZNF474
ADCY1	TMEM2	C5orf49
RAI1	FLRT3	PACRG
CPLX1	ZNF324	SSC4D
FRS3	TENM4	CR2
HPSE	NKX2-8	HGSNAT
PPARGC1A	SLC13A4	CARNMT1
ADCY3	GCNT2	C9orf135

BTG3	OSTF1	CRX
CLP1	GJA3	MACROD2
CCNI	PRTG	NRSN1
RBPMS	GRB10	ITLN2
B4GAT1	HCRT	LDLRAD3
TPPP	HYAL1	A2ML1
DNAJB4	ITPR2	CLDN19
PKIG	LAMB3	C1orf210
PSIP1	PRICKLE3	CTNND2
ADCY6	NFIB	PROM2
AKAP2	NKX2-2	CTSB
DUSP10	PAH	CTSH
PDCD10	GALNT9	MARVELD2
EGLN3	ATP6V0A4	MBOAT1
ZHX1	HERC5	RASEF
XPOT	CDK18	DENND5B
MGAT4A	LARS	TMEM30B
SCRG1	ERAP1	FNDC7
MGLL	PLP1	DCT
CHRNA4	PMAIP1	UGT3A2
PIK3IP1	PPARA	NIM1K
CCDC85A	GIPC2	FAM151B
RNF157	TTC12	C6orf118
CGAS	NMRK1	GSDME
DDIT4L	SOX6	ADAMTS19
FAM210B	CDC42BPG	DIO2
PHACTR3	LANCL2	ABAT
FAM129A	KIAA1217	DUSP6
WDR17	PROS1	ELF3
DACH2	C1GALT1	LYRM9
DBX1	RGL3	ENO1
BTBD11	PXMP2	STK32A
ANKRD9	SIGIRR	EPHA1
NIPA1	PLEKHA2	SLC44A5
ZNF813	PCIF1	TRIML2
JOSD2	RAB17	ERBB4
ZNF428	SGK1	F2RL1
TYW3	SH3GL2	FABP6
COL1A2	SPINK2	FAT2
FITM2	TLR3	ZNF25
DUSP15	TPMT	DOK6
FBLN7	ZIC3	PRR15
CLHC1	ZNF90	FGF2

AP1S3	ZNF165	FGF9
C4orf33	RNF103	FGF14
UBE2QL1	MALL	FHL1
HINT3	CALB1	PUF60
CLDN4	CLMP	ANKRD6
CLDN3	ZNF436	FOXG1
DCAF12L1	DUSP16	RUFY3
CRIP2	PLA2G12A	SEPHS1
PNCK	TIGD6	DAAM1
RNF32	ESPN	FLI1
ZFP28	SNX25	DTX4
NOL4L	ANKRD27	SEL1L3
RBBP8NL	CASP8	MTCL1
STK35	HPDL	MCF2L
B3GLCT	BHLHE40	WWC1
TTC7B	PLPP2	SATB2
FAM81A	PDE5A	DOCK9
TOM1L2	CCK	NR5A2
CFAP52	SEC16B	ACKR1
CST3	BTBD6	ZDHHC23
CCBE1	CLDN9	RNF144B
APCDD1	PCED1B	GABRB3
SAMD11	LRRFIP1	GABRG3
CTGF	AKAP6	MGAT4C
ZNF362	IGDCC3	GALNT3
NOL4L-DT	SDC3	CNRIP1
FAM19A4	SGSM2	GAP43
EFHB	ARNT2	TES
CYB5A	CDC25B	MDGA1
SAMD3		GPR160
ABCA13		CACNG4
ATP6V0E2		SMPDL3B
TDRP		SFN
FREM1		GPLD1
PRUNE2		SLCO4A1
FAM122C		RIIAD1
GRASP		GPR37
TMEM92		C8orf31
TTLL9		GRB14
AES		GRIA1
ZNF467		MAGEH1
GLIS3		TAGLN3
ADAMTS17		SLC40A1

DIAPH2		RAX
DLX5		HAS2
DMD		NRG1
RCAN1		HK1
DSG2		AOX1
APLNR		HSD17B4
EDA		HSPA2
LPAR1		HTR3A
PHC2		TNC
EFNA5		RTL5
EFNB2		NEXMIF
CELSR2		OTOG
EGFR		NCKAP5
EGR2		MACC1
AHR		RILPL1
AMN1		SLCO4C1
DTX3		IRF6
METTL7B		CCDC172
ELAVL4		FAM228B
CADM4		KCNJ8
KRTCAP3		KRT8
TMEM17		KRT18
ARHGAP27		CCDC184
ENO2		KRT19
ENO3		HES5
NRK		IER5L
DMTN		LAMC2
STOM		LCK
EPHB3		LDHA
FBL		LMO1
ETFB		LMO7
MECOM		RAB19
F10		LPL
AMER2		LTBR
UNC5B		LY75
HYLS1		EPCAM
DDIAS		MAP1B
FAM124A		ARSD
OAF		MAT1A
RASGEF1A		MET
SPATA13		CITED1
AK9		MSH2
NT5DC1		MST1R

KDM1B		MYCN
VWDE		NOVA1
HS3ST5		PRKN
SLC29A4		F11R
FEN1		YARS2
FGFR1		CRYL1
ZFP30		PLCE1
ADGRL1		HOOK1
KIFAP3		PDE4D
ELL2		NT5DC3
RIMS1		ENPP1
MYT1L		PADI3
CLASP2		ATP8B1
KDM6B		KCNK10
NACAD		GPR173
SEPT6		PON2
LPIN1		SDK2
FLNC		L1TD1
RFTN1		POU5F1
KANK1		ESRP1
GSE1		MARC2
FLT1		DET1
SULF1		PPP2R5A
ARHGEF9		TRPV6
UFL1		PLXNA3
KCNH3		TSR1
ABCA5		MCTP2
TTC9		MBNL3
ZNF281		PRKCH
MAPK8IP2		PRKD1
LPAR3		MYO5C
DDAH1		MAPK10
CDC42EP4		KLHL4
KCNE5		PRLR
SSBP2		SPIRE1
FLRT1		CA10
ABCA4		RGMA
SLC17A8		SLC24A3
FUCA2		NLGN4X
SFTA3		KCTD16
GABRG2		CACHD1
WDR72		ADGRB3
GAD1		PTPRN2

KLHL34		PXN
SHC2		JAM2
SLC39A6		MID1IP1
GALC		LGR6
TRIM58		BCL2
HSPA12A		RFX2
ZNF521		RFX4
WWTR1		RGR
KANK2		RTN1
DNAH1		BCORL1
CLIP3		SCNN1A
ACOT11		PKNOX2
SIPA1L1		TNN
LRRTM2		TMBIM1
ERC2		GALNT17
GPSM1		NDST4
ZBTB20		C6orf132
ZNF337		BMI1
LHX6		ST6GAL1
GCNT1		SIX3
CHIC2		SLC1A4
AMFR		RASL11B
GFRA1		SMARCD2
RNF11		SOD3
CLUL1		SOX5
FAM155B		TCF7L2
GPR162		TFAP2A
APEX2		TGIF1
TOX3		TNNT2
PCSK1N		FAM160A1
CECR2		COL14A1
GNAO1		VIM
SELENOH		VIPR1
BCL9L		MPPED2
DPY19L2		SLC30A4
ZNF843		FZD5
ANGPT1		C1orf116
GPR17		SCRN3
TTLL6		ARSJ
CAVIN1		C3orf52
PRR19		VEPH1
CCDC141		JADE1
NKIRAS1		GRHL2

EPHA6		GREB1L
CYP4V2		STMN4
ANK1		COL21A1
ANK2		DPF1
ASTE1		NRIP1
HIPK2		CAPN6
GRM1		DYSF
GPSM2		INHBE
ALG6		HMCN1
NXPH1		TTC29
ANXA3		RASSF4
HLA-DOA		NCALD
HMX1		SOX14
HNRNPF		FYTTD1
APBA1		BRSK1
APBA2		MAP3K21
APBB1		NKX6-2
HSPA1A		PPFIBP2
APLP1		TOX2
DNAJB1		SLC43A1
APOB		PIK3R3
CCDC137		ZC3H12C
BRINP3		GALP
ID1		TMEFF1
ARSI		VAMP8
IDE		TNFRSF11A
IDUA		TNFRSF10A
GPR149		NRP2
TUBB2B		KAT2B
AMIGO2		NAV3
IGF2		NAV1
ZNF713		NAV2
WDR86		FAM181A
IL11RA		SEMA5A
IMPA2		MAP7
INSM1		PAPSS2
IRF1		SYT7
IRF4		CLDN10
KCND3		MAGI1
ANKRD36		LRAT
KCNH2		CAPN13
RAB15		ADAMTSL1
KCNJ9		B3GNT7

KCNK1		TJP2
KCNN1		NTN1
KCNN2		TP53INP1
KIFC3		PCYT1B
ENO4		EEF1E1
C11orf96		BCAR1
LINC01551		ZNF516
C16orf87		NOS1AP
GLTPD2		RIPOR2
LAMA4		LRBA
LAMB1		SNAP91
ABLIM1		CDH1
FSIP2		
CRIP3		
LOXL2		
TACSTD2		
SMAD3		
MAN2A2		
MAP1A		
MAP2		
MAT2A		
CHST6		
ART3		
MEIS2		
MEST		
KITLG		
MICB		
MITF		
AFF1		
MME		
ASL		
MPP3		
AARD		
MSI1		
MYH7		
MYO5B		
NCAM2		
NELL2		
ATP1A2		
NFIA		
NOS2		
NOVA2		
RNF165		

OCA2		
CLEC18B		
C18orf32		
OTX2		
P2RX3		
DEF6		
PAK3		
PNPLA8		
PAPPA		
PAX6		
GMNN		
ATL1		
SCCPDH		
TFB1M		
SBDS		
MYO15A		
LEF1		
PLEKHO1		
PCSK1		
TAOK3		
PDE1A		
PDE4B		
TDP2		
NBAS		
YPEL5		
CHMP3		
ENPP2		
CYB5R2		
SIX4		
PLCB2		
BCL11A		
IL17D		
NUP54		
FGFRL1		
CYCS		
SLC38A2		
NLGN3		
RETREG1		
RBM47		
POU2F1		
MAGEL2		
EPB41L4B		
FBXL19		

MANSC1		
GRAMD1C		
NUP62CL		
MTMR10		
RNF125		
PIGX		
TMEM132A		
PARPBP		
PID1		
OXR1		
SOBP		
SLFN12		
PPP2R1B		
KIAA1551		
DPPA4		
KLHDC8A		
ADI1		
PHF10		
ACOXL		
PPP3CA		
AGPAT5		
CAMK2N1		
CSGALNACT2		
ASIC4		
SVOP		
CHRNA9		
CARMIL1		
TTC27		
SMPD4		
KRBOX4		
SYBU		
H2AFJ		
WSB2		
ERBIN		
SMG9		
ANKH		
PRNP		
FMN2		
TCEAL7		
FSTL5		
DPYSL5		
ANKS1B		
MEIS3		

OLFML3		
STOX2		
PRDM8		
AVEN		
CAMK1D		
SALL4		
VANGL2		
SNX14		
C3orf14		
PPM1H		
PLEKHG1		
MTUS1		
CGN		
MIB1		
IFT80		
EBF4		
USP28		
BEND3		
ZSWIM6		
PTPN3		
BRINP2		
PTPN14		
ZNF410		
CADM3		
PTPRD		
PTPRE		
PTPRM		
PTPRN		
PTPRR		
OVOL2		
RRAGD		
RAB27B		
ENPP5		
RARA		
RARB		
RBMS1		
RET		
RGS4		
RNASE4		
AASDHPPT		
BCL7A		
CELF5		
RYR2		

BDNF		
BFSP1		
SCN1A		
BID		
CXCL5		
PIEZO2		
NAPB		
PRDM14		
C19orf33		
ADGRL4		
TINAGL1		
SFRP2		
ZNF862		
ARAP3		
SGCB		
CPEB1		
SHC1		
C16orf58		
FBXL17		
ISL2		
ST3GAL3		
BCL11B		
REEP1		
BMP4		
BMP7		
SLC15A2		
SLC16A1		
SLC18A1		
SLIT1		
SMARCA2		
CAPRIN2		
ZBTB10		
KCTD14		
SMARCD3		
SNRPN		
SOAT1		
BNIP3L		
SOX1		
BOK		
HSPA13		
TARBP1		
ZEB1		
BTG1		

TEK		
TFAP2C		
NR2F2		
TGFBR2		
THPO		
TIMP2		
NKX2-1		
TLE4		
TLR5		
C1S		
TPBG		
TPM1		
TRAF3		
TRAF5		
TTC3		
TUBA4A		
VLDLR		
VRK2		
WRN		
LDLRAD4		
MPPED1		
MKRN3		
ZNF215		
ZNF217		
ZYX		
COLEC11		
ST8SIA4		
ALDH5A1		
TTC13		
SPAG16		
ACSS3		
CCNJL		
SRD5A3		
BEND5		
PLEKHF2		
ZDHHC14		
MORC4		
ZBBX		
ZNF385D		
AGMAT		
EFCC1		
LPCAT1		
ELOVL7		

WWC2		
BICC1		
BBOF1		
NR4A3		
ZNF703		
CHD9		
FHOD3		
CCDC92		
SP6		
KCNIP4		
SLC25A16		
SLC19A3		
AKNA		
PBX4		
ZFP91		
TMPRSS5		
IFT88		
AP3B2		
ST8SIA2		
GPR63		
ITFG1		
APOLD1		
LBH		
SLCO5A1		
VANGL1		
ZNF93		
DGCR6		
DRC3		
CD99L2		
TM2D2		
ULK1		
USP44		
EVA1A		
SLITRK6		
PSD2		
TMEM175		
RASAL1		
PHYHIPL		
LCOR		
COL25A1		
TNRC18		
CAT		
LNX1		

USP30		
KLHL22		
ZDHHC12		
ZSCAN10		
RGS5		
ATOH8		
RAB2B		
CGNL1		
CEP19		
TCEAL3		
PKP4		
YBX3		
CPZ		
RGS8		
DCLK3		
TNKS1BP1		
RRP1		
CHRD		
SLC4A4		
ACTN1		
SERPINH1		
NOL4		
CBR1		
DLK1		
FGF17		
ST3GAL5		
CCNA1		
AP1M1		
WASF1		
RSPH1		
CCNE1		
KALRN		
NAT1		
BRSK2		
ANGPTL1		
SLC16A5		
AIFM1		
LONRF1		
ZNF804A		
TTC5		
ZMYM5		
CDHR1		
MSC		

UBE2L6		
BBIP1		
BICDL1		
TMEM169		
DNER		
PTER		
TRIP13		
MMGT1		
ARMC6		
CER1		
NRXN3		
QKI		
STX8		
CREB5		
CELSR1		
SNCAIP		
NUP155		
ARHGEF10		
UBE3C		
ULK2		
KIAA0408		
STARD8		
SUSD6		
SCRN1		
DNAJC6		
TRIM14		
ZBTB24		
RABGAP1L		
AMMECR1		

Table S5: ***Pathway analysis of DEGs from days 35 and 45.*** Significant DEGs from the day 35 and 45 comparison (|FC|>2) were input into DAVID Bioinformatics Resources (v6.8). Pathway analysis (GO: Biological process) yielded 10 significant pathways (FDR<0.05, -log10(Benjamini-Hochberg>3)). Count refers to number of genes from dataset found in pathway, % genes from list refers to percent of genes in pathway from dataset provided.

Term	Count	%	-log10 (Benjamini-Hochberg)	FDR
GO:0007601~visual perception	84	3.166227	18.34213	1.57E-19
GO:0050896~response to stimulus	34	1.281568	9.498997	2.19E-10
GO:0009653~anatomical structure morphogenesis	34	1.281568	4.066283	8.89E-05
GO:0030049~muscle filament sliding	20	0.753864	3.994986	1.40E-04
GO:0042391~regulation of membrane potential	29	1.093102	3.696164	3.47E-04
GO:0007155~cell adhesion	102	3.844704	3.602189	5.18E-04

GO:0030198~extracellular matrix organization	54	2.035432	3.590927	6.20E-04
GO:0045944~positive regulation of transcription from RNA polymerase II promoter	187	7.048624	3.524445	8.26E-04
GO:0035725~sodium ion transmembrane transport	27	1.017716	3.059333	0.002711
GO:0008217~regulation of blood pressure	25	0.942329	3.02551	0.003257
